# Treatment with the direct oral anticoagulants (DOACs) Apixaban and Rivaroxaban associated with significant worsening of behavioural and psychological symptoms of dementia (BPSD)

**DOI:** 10.1192/bjo.2021.346

**Published:** 2021-06-18

**Authors:** Kim Porter, Stephen De Souza, Iain Hargreaves, Rebecca Goddard

**Affiliations:** 1 Somerset NHS Foundation Trust; 2 University College London; 3 Avon and Wiltshire Partnership Trust

## Abstract

**Objective:**

To demonstrate the increasing evidence for an aetiological role of cerebral mitochondrial dysfunction in neuropsychiatric disorders.

To raise awareness of the importance of frontline staff partaking in post marketing surveillance of medications.

**Case report:**

We report the cases of two patients who developed worsening BPSD, coinciding with starting the factor Xa inhibitor DOAC medications Apixaban and Rivaroxaban respectively. Both patients required detaining under the Mental Health Act (MHA). Their symptoms improved significantly, within two weeks, on switching to alternative anticoagulant therapies and they were both discharged from the acute psychiatric ward.

**Discussion:**

Frontline healthcare staff in acute settings and the community manage a heavy workload. It is all too easy to overlook potential neuropsychiatric drug side effects, especially if they are not clearly listed. They may be easily missed amongst older patients and wrongly attributed to dementia.

Rivaroxaban is structurally related to the antibiotic Linezolid which has been reported to cause mitochondrial toxicity. Pre marketing In vitro studies concluded the risk of mitochondrial toxicity associated with this anticoagulant to be low. However a more recent in vitro study, using rat kidney mitochondria, reported evidence of mitochondrial swelling and a collapse of the membrane potential following exposure to low doses of Rivaroxaban. The effect of Apixaban, which is structurally related to Rivaroxaban, has yet to be investigated on mitochondrial function.

Recent research supports not only an association between reduced cerebral mitochondrial function and neuropsychiatric symptoms and disorders, but also the aetiological role it may play.

There is a need for a far greater awareness and understanding of the potential cerebral mitochondrial toxicity of drugs commonly prescribed to our older populations.

**Conclusion:**

Cerebral mitochondrial toxicity can have a significant impact on the health and well-being of patients.

Older patients are particularly prone to experiencing neuropsychiatric side effects that may not have been apparent during preclinical trials.

Development of a rating scale of drugs that are potentially less toxic to cerebral mitochondria could inform national prescribing guidelines and enable safer treatments to be offered to older people, reducing the likelihood of them experiencing apparent behavioural and psychological symptoms of dementia.

